# Monitoring the patient–ventilator asynchrony during non-invasive ventilation

**DOI:** 10.3389/fmed.2022.1119924

**Published:** 2023-01-19

**Authors:** Federico Longhini, Andrea Bruni, Eugenio Garofalo, Simona Tutino, Luigi Vetrugno, Paolo Navalesi, Edoardo De Robertis, Gianmaria Cammarota

**Affiliations:** ^1^Anesthesia and Intensive Care, Department of Medical and Surgical Sciences, Magna Græcia University, Catanzaro, Italy; ^2^Department of Anesthesia and Intensive Care Unit, SS Annunziata Hospital, Chieti, Italy; ^3^Department of Medical, Oral and Biotechnological Sciences, “Gabriele D’Annunzio” University of Chieti-Pescara, Chieti, Italy; ^4^Anaesthesia and Intensive Care, Padua Hospital, Department of Medicine, University of Padua, Padua, Italy; ^5^Department of Medicine and Surgery, University of Perugia, Perugia, Italy

**Keywords:** non-invasive ventilation, patient–ventilator asynchrony, ventilator waveforms, diaphragm electrical activity, pressure support ventilation (PSV), neurally adjusted ventilatory assist (NAVA), proportional assist ventilation (PAV)

## Abstract

Patient**–**ventilator asynchrony is a major issue during non-invasive ventilation and may lead to discomfort and treatment failure. Therefore, the identification and prompt management of asynchronies are of paramount importance during non-invasive ventilation (NIV), in both pediatric and adult populations. In this review, we first define the different forms of asynchronies, their classification, and the method of quantification. We, therefore, describe the technique to properly detect patient**–**ventilator asynchronies during NIV in pediatric and adult patients with acute respiratory failure, separately. Then, we describe the actions that can be implemented in an attempt to reduce the occurrence of asynchronies, including the use of non-conventional modes of ventilation. In the end, we analyzed what the literature reports on the impact of asynchronies on the clinical outcomes of infants, children, and adults.

## Introduction

Patients with Acute Respiratory Failure (ARF) may benefit from different oxygenation or ventilation supports (1, 2). In patients affected with moderate to severe forms of ARF, including cardiogenic pulmonary edema and acute-on-chronic respiratory failure, non-invasive ventilation (NIV) plays a major role (1). However, NIV is affected by a certain percentage of treatment failure, requiring mostly orotracheal intubation and institution of invasive mechanical ventilation (3).

Behind the type and severity of ARF, worsening of gas change, respiratory distress, hemodynamic instability, or neurological deterioration, NIV may also fail because of the patient’s intolerance to the treatment (3, 4). Among the reasons for treatment intolerance, there is a type of interface applied to the patient, the presence of massive air leaks, and the occurrence of patient**–**ventilator asynchronies (5).

Patient**–**ventilator asynchrony is still a major issue during NIV in neonatal, pediatric, and adult patients. In particular, patient**–**ventilator asynchrony significantly contributes to increasing the work of breathing (6, 7), as well as generating discomfort (8, 9). Although mechanisms behind these phenomena are well described (10–13), the impact of patient**–**ventilator asynchronies on clinical outcomes is still debated.

After defining the varying types of asynchronies, we aim to review the literature of the last 30 years about patient**–**ventilator asynchronies occurring during NIV in neonatal, pediatric, and adult patients with ARF. We aim to focus on the quantification, detection, management, and impact of asynchronies on the clinical outcomes of patients undergoing NIV.

## Materials and methods

### Search strategy for studies selection

The following search strategy was launched in PubMed on 10th November: ((“1992”[Date – Publication]: “2022”[Date – Publication]) AND (“patient**–**ventilator asynchrony” OR “patient**–**ventilator interaction” OR “ineffective effort” OR “wasted effort” OR “autotriggering” OR “auto-triggering” OR “double triggering” OR “premature cycling” OR “delayed cycling”)).

**FIGURE 1:**
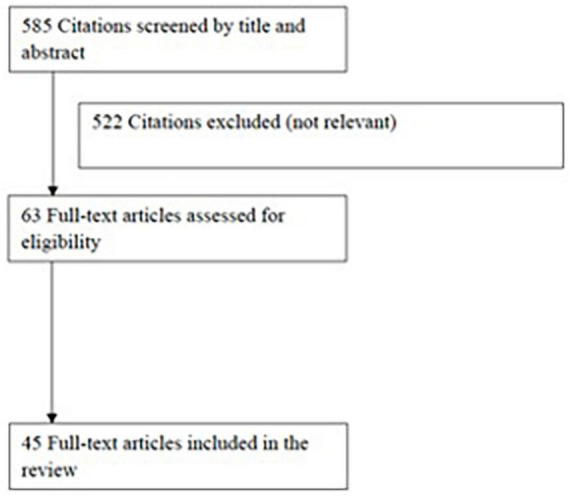
Study flow diagram according to the preferred reporting items for systematic review and meta-analysis protocols recommendations.

After retrieving all references in the published reviews to identify other studies of interest missed during the primary search, two authors independently checked all the articles and selected those enrolling neonatal, pediatric, and adult patients with ARF undergoing NIV, published between 1 January 1992 and 1 November 2022 in the English language. In case of disagreement, the expert opinion of a third examiner was requested for a conclusive decision. Case reports, review articles, editorials, and studies available only in abstract forms were excluded (Figure 1). Of the 585 searched records, 45 studies were included in the manuscript and their references were retrieved for further titles.

### Definitions

**FIGURE 2:**
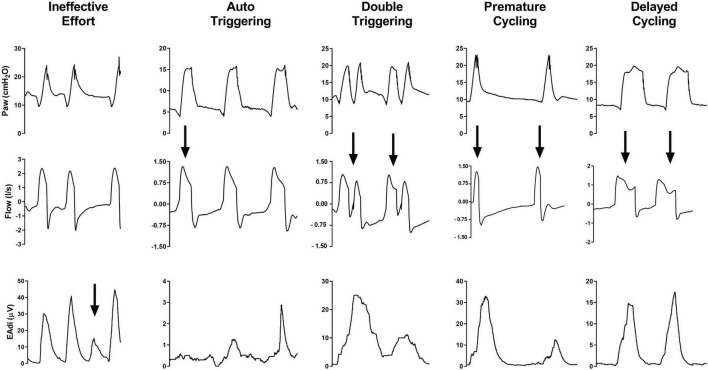
From top to bottom, waveforms of airway pressure (Paw), flow, and electrical activity of the diaphragm (EAdi) are depicted for each type of patient–ventilator asynchronies. Arrows highlight the asynchronous events.

Asynchronous events are the lack of coordination between the respiratory activity of the patient and the mechanical assistance of the ventilator. During NIV, patient**–**ventilator asynchronies have been classified as (1) major (ineffective triggering, auto-triggering, and double-triggering) and (2) minor (premature or anticipated cycling, prolonged or delayed cycling, and triggering delay), depending on the extent of the disturbance of coordination (14). An example of each type of asynchrony is depicted in Figure 2.

Ineffective triggering, also known as ineffective or wasted efforts, is defined by a patient’s inspiratory effort not assisted by the ventilator. This asynchrony may appear during both the expiratory phase of the ventilator and the inspiratory ventilatory assistance. The possible underlying mechanisms are recognized to be weak respiratory drive and/or effort, a high intrinsic positive end-expiratory pressure (PEEPi), and an excessively low ventilator trigger sensitivity (13, 15–18).

Auto-triggering consists of a mechanical insufflation not triggered by any inspiratory effort of the patient. This type of asynchrony is commonly triggered by disturbances in airway pressure and/or flow or by air leaks, which are wrongly sensed as triggering efforts (15, 19). Therefore, their occurrence depends primarily on trigger type, sensitivity, and the ability of the ventilator to compensate for air leaks (20).

Double-triggering is characterized by one single patient inspiration supported by two mechanical cycles separated by a very short expiratory time (<30% of the mean inspiratory time) (15). The interruption of the mechanical insufflation before the completion of the patient’s effort generates a second triggered mechanical insufflation, after a brief exhalation phase (8, 15).

Premature cycling is a form of patient**–**ventilator asynchrony characterized by an interruption of the ventilator insufflation anticipating the patient’s effort termination; whereas, in the case of delayed cycling, the mechanical assistance is longer than the patient’s effort and it extends into the patient’s own (neural) expiration. Premature cycling is more frequent in patients with Acute Respiratory Distress Syndrome (ARDS) (21, 22) and it may result in double triggering (23), whereas delayed cycling occurs more frequently in obstructive conditions (16). During NIV, delayed cycling is most commonly induced by air leaks which prevent the achievement of the expiratory trigger threshold and insufflation cycling-off (24).

## Detection of asynchronies

The rate of asynchrony is commonly measured by the Asynchrony Index (AI%), defined by the ratio between asynchronous breaths and the overall breath count, that is, the sum of ventilator cycles and non-triggered breaths expressed as a percentage (25). In patients undergoing invasive mechanical ventilation, an AI% of ≥10 is associated with worsened clinical outcomes (15, 25, 26). On the opposite, AI% values of ≥10 in patients undergoing NIV are associated with poorer comfort reported by the patients, but not with intubation rate, length of stay in ICU, or mortality (8). Therefore, whenever the AI% value is ≥10, the physician should implement actions to reduce the rate of asynchronous events (refer to the following text).

Sinderby et al. also proposed an automated and standardized method to quantify asynchronies, the so-called NeuroSync Index (27). This index is based on the assessment and monitoring of the Electrical Activity of the Diaphragm (EAdi), which requires a dedicated catheter connected to a specific ventilator to acquire the diaphragmatic signal, and an off-line analysis of the ventilator waveforms to address the rate of asynchronies. The NeuroSync Index was shown to be reproducible and correlated with a manual analysis by experts (27).

### Neonatal and pediatric patients

When high-flow oxygen therapy fails, NIV is considered the gold standard treatment in newborns, infants, and pediatric patients affected by ARF (28–30). Patient**–**ventilator asynchrony is a major challenge in non-adult patients and it is commonly evaluated with the adjunctive EAdi signal, to monitor the diaphragmatic signal and respiratory effort (10, 11, 31). In 35 newborns and children undergoing NIV in Pressure Support Ventilation (PSV) mode, Vignaux et al. reported that the median AI% was 65 (32). Ineffective efforts, auto-triggering, and premature cycling were the most common types of asynchrony. The authors also reported that, after adjusting and optimizing the ventilator settings, the median AI% significantly decreased to 40 (32). Extremely premature infants undergoing conventional modes of NIV can be characterized by even higher median AI% up to 86%, as recently reported (33). In the pediatric population, it has been demonstrated that the use of adjunctive signals, such as the EAdi, improves the ability of pediatric intensivists to detect ineffective efforts and auto-triggering (34).

### Adult patients

In adult patients, patient**–**ventilator asynchronies have been evaluated with several methods, such as the observation of waveforms on the ventilator screen (14), dedicated algorithms (35), or additional signals (i.e., EAdi, esophageal, or transdiaphragmatic pressure) (36, 37).

Visual inspection of ventilator waveforms is the most common method adopted in routine clinical practice. In fact, this method does not need any placement of additional catheters, which can be considered difficult to be positioned and a source of further discomfort for the patient and air leaks. However, a multicenter study showed a very low sensitivity by expert and non-expert physicians in detecting asynchronies during NIV through a helmet or face mask by the sole ventilator waveform inspection (14). Worth remarking, the rate of correct detection was inversely related to the prevalence of asynchronies (14).

Mulqueeny et al. developed an automated algorithm to detect ineffective efforts, such as expiratory flow perturbation without any ventilatory support, and double-triggering, as two mechanical ventilatory inspiratory cycles separated by less than 500 ms (35). In 10 patients undergoing NIV in PSV mode, this algorithm showed a specificity of 95.1% in the detection of asynchronies. However, this algorithm has the inner limitation to detect only ineffective efforts during expiration and double triggerings (35).

The NeuroSync Index, proposed by Sinderby et al. (27), is another automated algorithm tested during NIV in 12 patients with acute-on-chronic respiratory failure (38). NeuroSync Index ensured a proper detection of wasted efforts, triggered delays, and cycling-off errors during PSV delivered by a dedicated NIV ventilator or an ICU ventilator equipped with software for air-leaks compensation, and by non-invasive Neurally Adjusted Ventilatory Assist (NAVA) (38).

As mentioned earlier, this algorithm requires the positioning of an EAdi catheter, which somehow increases costs and the use of a dedicated ventilator equipped for EAdi monitoring and NAVA ventilation. Therefore, this system has inner limitations which restrict its application in all centers.

More recently, the application of diaphragmatic ultrasonography has been proposed to recognize patient**–**ventilator asynchronies during invasive mechanical ventilation (39). This technique has also been tested in healthy volunteers undergoing NIV with induced asynchronies (40). This method comprises monitoring the diaphragm dome excursion or its thickening in the apposition zone, to define the presence of the patient’s respiratory effort (40). Diaphragm ultrasonographic imaging was then in real-time coupled with the ventilator waveforms to recognize and accurately identify asynchronies (40). To note, despite diaphragm ultrasonography could be considered an “easy to learn technique” (41), the need to visualize the airway pressure curve on the ultrasound machine screen limits its use in daily clinical practice (40). That said, whenever the ventilator waveforms will be screened on the ultrasound machines, this technique may potentially have a major role in the future to assess patient**–**ventilator synchrony.

Finally, Electrical Impedance Tomography, a tool for bedside functional imaging of the lung, has been applied in ARDS porcine model to study the “pendelluft” phenomenon in case of asynchronies with the ventilator (42). Besides this recent and experimental use, no studies have so far evaluated the aeration or lung ventilation distribution in patients undergoing invasive mechanical ventilation or NIV with severe patient**–**ventilator asynchronies.

## Management of asynchronies

### Neonatal and pediatric patients

In neonatal and pediatric patients, the management of patient**–**ventilator asynchrony is of paramount importance. Since non-adult patients have a respiratory rate of up to 50 breaths/min, an optimal patient**–**ventilator synchronization could better unload the diaphragm (32, 43, 44).

In the case of patient**–**ventilator asynchronies, the physician should first assess the ventilator settings and the applied interface. In fact, by adjusting the expiratory trigger settings during PSV, patient**–**ventilator synchrony improves (32). In addition, the presence of considerable unintentional air leaks also affects patient**–**ventilator synchrony. Therefore, a change in the type of interface or adjustment of its position should be considered (45). However, if these actions fail to reduce asynchronies, non-conventional modes of ventilation can be considered. NAVA is a non-conventional mode of ventilation driven by the EAdi signal that delivers inspiratory assistance proportionally to EAdi, which is the closest recordable signal of the patient’s central respiratory drive (31). In particular, non-invasive NAVA was shown to guarantee optimal synchronization despite large air leaks or weak respiratory efforts (32, 46, 47).

### Adult patients

Unintentional air leaks are the most important source of asynchrony during NIV in adults (8, 24). The presence of massive air leaks may generate a particular condition called “flow asynchrony”. In fact, flow asynchrony is defined as a ventilator flow output not coinciding with the patient’s inspiratory flow demand (48). In intubated patients, flow asynchrony increases the work of breathing (49) and dyspnea (50). To contain the occurrence of flow asynchrony, it is essential to optimize the flow delivery by adjusting the rise time, to apply NIV with a dedicated ventilator equipped with air leaks compensating software, and to reduce intentional and un-intentional leaks (3, 51, 52).

Therefore, the choice of a proper interface, the adjustment of ventilator mode and settings, and the use of ventilators with air-leaks compensating software can reduce the occurrence of patient**–**ventilator asynchronies, including flow asynchrony (10, 11).

The choice of the NIV interface and assessment of its positioning should be one of the first actions to implement in the case of patient**–**ventilator asynchrony (3). When NIV is delivered through masks or mouthpieces, the amount of air leaks is substantially different, and the higher the leaks, the higher the rate of asynchronies (53). As compared to invasive mechanical ventilation, both the mask and helmet as NIV interfaces increase the occurrence of asynchronies (54). Several studies have reported that the helmet generates a higher rate of asynchrony, compared to the mask (54, 55). Since the helmet has inner drawbacks related to the high inner volume and upward displacement during ventilator insufflation, a new generation of the helmet has been developed to improve the pressurization and patient**–**ventilator interaction (56, 57). As compared to the conventional helmet, the new one reduces the inspiratory trigger delay, increases the time of synchrony between diaphragm activity and ventilator assistance, and overall improves comfort (58). However, the recorded asynchronous events are similar between interfaces (58). Physicians should also minimize the number of air leaks because these events caused discomfort by themselves and are associated with asynchronous events (8).

The adjustment of ventilator settings and mode is another variable that could be corrected in case of patient**–**ventilator asynchrony during NIV. Among settings to be checked, a too-high inspiratory pressure is associated with AI% >10% (8). Furthermore, the cycling-off criterion should also be addressed and set with an individualized approach, to optimize synchronization with the ventilator and to avoid the “hung-up” phenomenon (24, 59).

In addition, the use of proportional modes of ventilation should also be considered, such as Proportional Assist Ventilation (PAV) or NAVA. PAV was shown to be comfortable and tolerated in patients with moderate ARF (60, 61), which may be in part attributable to synchrony. Another study has recently compared PAV with PSV in 15 patients with exacerbated COPD (62). PAV did not improve patient**–**ventilator interaction; in addition, the use of PAV+, a development of PAV, induced the runaway phenomenon (62), which may contribute to asynchrony (63). Indeed, PAV + requires a closed system without air leaks, making this mode no longer used during NIV (63).

In particular, while PAV requires that the physician set the assistance parameters (i.e., flow and volume assist) based on the respiratory mechanics of the patient, PAV+ has implemented software that continuously monitors the patient demand by measuring flow and volume every 5 msec during and by implementing short end-inspiratory occlusions. The physicians are asked to set only load-adjustable gain factors, and the ventilator would proportionally deliver inspiratory support based on the equation of motion of the respiratory system (64). Therefore, while air leaks would not impair the functioning of the former PAV mode, PAV+ requires a closed system to assess the flow and volume and to perform end-inspiratory occlusions (64).

On the contrary, several studies have investigated and proved that NAVA can efficiently optimize patient**–**ventilator synchrony during NIV delivered either by helmet (36) or by mask (65–67). More recently, a specific setting of NAVA (defined as Neurally Controlled Pressure Support) has been described during NIV through helmet (68, 69) and mask (70). Neurally Controlled Pressure Support significantly improved patient**–**ventilator interaction and synchrony, compared to PSV.

Third, the use of ventilators equipped with software capable to detect and compensate for air leaks significantly improves patient**–**ventilator interaction and synchrony (51, 71). Of note, Carteaux et al. did not confirm that the presence of NIV software reduced the occurrence of asynchronous events in ICU ventilators; however, the use of dedicated NIV machines significantly improved patient**–**ventilator synchrony (72).

## Impact of asynchronies on the patients’ outcomes

### Neonatal and pediatric patients

In an attempt to mitigate the possible effects of patient**–**ventilator asynchronies on clinical outcomes in neonatal and pediatric patients, several studies have compared NAVA with conventional modes of ventilation during NIV.

In a randomized crossover trial, Lee et al. randomized 15 preterm infants to receive NIV in NAVA and PSV modes (73). The authors reported that NAVA reduced the work of breathing and improved patient**–**ventilator synchrony, as compared to the conventional mode of NIV, even in the presence of large air leaks (73). In keeping with Lee et al. (73), Gibu et al. included eight preterm infants to receive NIV in NAVA or PSV modes (74). Infants appeared to be more comfortable during NAVA, as compared to conventional modes. However, no other clinical outcomes have been reported by both studies (73, 74). A recent systematic review with meta-analysis showed that NAVA and conventional modes of ventilation are characterized by similar NIV failure rates, but it could not determine if NAVA would prevent the worsening of respiratory failure (75). One recent randomized controlled trial has reported that NAVA ameliorated patient**–**ventilator synchrony; however, no differences were recorded with respect to vital parameters (i.e., heart rate and respiratory rate), comfort, apneic events or desaturations, and bradycardias (33). Another recent randomized controlled trial showed that NIV in NAVA modes reduced the occurrence of post-extubation respiratory failure in preterm infants, as compared to Continuous Positive Airway Pressure (CPAP) (76). It should be noted that CPAP does not require interaction with inspiratory pressurization of a ventilator, and this result cannot be associated with a reduction of asynchronies rate.

A physiologic crossover study demonstrated that NAVA reduces the asynchronies rate with the ventilator, and also in infants, it is a feasible and safe mode for NIV and well-tolerated by the patients (77).

In a randomized crossover study, 18 children with mild ARF received NIV in NAVA or PSV modality. The study demonstrated that NAVA is a feasible and safe mode of NIV and it reduces the occurrence of asynchronies; however, given the study design, no data are available on major clinical outcomes (78).

In addition to the large amount of data suggesting that NAVA improves patient**–**ventilator interaction and some minor physiological outcomes, no randomized controlled trials have so far investigated the impact of patient**–**ventilator asynchronies on major clinical outcomes, such as the duration of mechanical ventilation, ICU, or hospital lengths of stays and mortalities in the pediatric patients.

### Adult patients

As mentioned earlier, the presence of patient**–**ventilator asynchronies may impair the tolerance and comfort of the patient to NIV, leading to treatment failure (3, 4, 8, 9).

AI% values of ≥ 10% significantly reduce the comfort and NIV tolerance in 60 patients who are critically ill (8) and another population including 69 acute patients undergoing NIV through oral-nasal masks (79). Proportional modes of ventilation such as NAVA have been also investigated in this regard and shown to reduce the occurrence of asynchronies (80). In a study by Schmidt et al., NAVA and PSV were compared with a cross-over design, also combining the presence or not of software for air-leaks compensation. Although NAVA improved patient**–**ventilator interaction and synchrony, comfort was not different between modes of ventilation (66). On the contrary, Neurally Controlled Pressure Support was demonstrated to enhance the pressurization and triggering performance, while guarantying optimal patient**–**ventilator synchrony during NIV through helmet (68, 69) and mask (70). In these settings, Neurally Controlled Pressure Support improved patients’ comfort with NIV (68–70).

Behind comfort improvement, no differences in mortality rate or ICU length of stay were detected between patients with or without an AI% value of ≥10% by Vignaux et al. (8). Another observational study has recently compared a cohort of 91 patients undergoing NIV in NAVA mode, with a historically and concurrently matched cohort of (134 and 202) patients undergoing NIV in PSV (81). After adjustment for confounders, NAVA did not improve the intubation rate, duration of NIV, and 90-day mortality, as compared to PSV (81). In the NAVA-NICE trial, 40 patients with acute exacerbated chronic obstructive pulmonary disease (COPD) were randomized to receive NIV through a mask in NAVA or PSV modes (82). Although reducing asynchronies, NAVA did not reduce the NIV failure rate, duration of NIV, or hospital mortality (82). Very recently, a large randomized controlled trial compared PSV and NAVA during NIV in a population of 100 patients with *de novo* ARF (83). In the overall population, this study did not demonstrate any difference in terms of NIV failure rates (30% vs. 32%, *p* = 0.83) and 28-day mortality rate (18% vs. 34%, *p* = 0.07) between NAVA and PSV, respectively (83). However, in the subpopulation of patients with exacerbated COPD, NAVA improved the 28-day survival rate, as compared to PSV (83). Worth mentioning, in patients with mild-to-moderate exacerbated COPD, if NIV is no more tolerated, a high-flow nasal cannula could be applied to avoid intubation, in the absence of further gas exchange worsening or respiratory distress (2, 84, 85).

It should be finally mentioned that NAVA can assure optimal patient**–**ventilator interaction and synchrony since the respiratory effort of the patient directly and proportionally triggers and leads the ventilator inspiratory support. Of note, NAVA requires an adjunctive cost for the dedicated catheter and proper training of physicians (31). To date, the extensive use of NAVA in all patients is not supported by the actual evidence of literature; however, well-defined patients may benefit from NIV through NAVA.

## Conclusion

Patient**–**ventilator asynchronies are common in both pediatric and adult patients during NIV. The detection of asynchronous events (even with adjunctive signals or automated software) is fundamental to implementing changes in ventilator settings and reducing their occurrence. Although high rates of asynchrony may affect the comfort of the patient and the success of the treatment, it remains to be demonstrated if patient**–**ventilator asynchronies may determine a worsened clinical outcome in patients undergoing NIV.

## Data availability statement

The original contributions presented in this study are included in the article/supplementary material, further inquiries can be directed to the corresponding author.

## Author contributions

FL, AB, EG, PN, ED, and GC provided substantial contributions to the conception and design of the work. AB, EG, ST, and LV participated in the acquisition, analysis, or interpretation of data for the work. All authors participated in drafting the work, whereas FL and GC further revised it critically for important intellectual content. All authors provided approval for publication of the content and agreed to be accountable for all aspects of the work in ensuring that questions related to the accuracy or integrity of any part of the work are appropriately investigated and resolved.
